# Development of a Multi-Layer Skin Substitute Using Human Hair Keratinic Extract-Based Hybrid 3D Printing

**DOI:** 10.3390/polym13162584

**Published:** 2021-08-04

**Authors:** Won Seok Choi, Joo Hyun Kim, Chi Bum Ahn, Ji Hyun Lee, Yu Jin Kim, Kuk Hui Son, Jin Woo Lee

**Affiliations:** 1 Department of Health Sciences and Technology, GAIHST, Gachon University, Incheon 21999, Korea; wschoi42@naver.com (W.S.C.); noirsky@naver.com (J.H.K.); 2 Department of Molecular Medicine, College of Medicine, Gachon University, Incheon 21999, Korea; cutemole@gmail.com (C.B.A.); totoro218@hanmail.net (J.H.L.); 3 Department of Plastic and Reconstructive Surgery, Gil Medical Center, Gachon University, Incheon 21565, Korea; pseugene@gilhospital.com; 4 Department of Thoracic and Cardiovascular Surgery, Gil Medical Center, Gachon University, Incheon 21565, Korea

**Keywords:** skin substitute, human hair keratinic extract, electrospinning, 3D printing, co-culture

## Abstract

Large-sized or deep skin wounds require skin substitutes for proper healing without scar formation. Therefore, multi-layered skin substitutes that mimic the genuine skin anatomy of multiple layers have attracted attention as suitable skin substitutes. In this study, a novel skin substitute was developed by combining the multi-layer skin tissue reconstruction method with the combination of a human-derived keratinic extract-loaded nano- and micro-fiber using electrospinning and a support structure using 3D printing. A polycaprolactone PCL/keratin electrospun scaffold showed better cell adhesion and proliferation than the keratin-free PCL scaffold, and keratinocytes and fibroblasts showed better survival, adhesion, and proliferation in the PCL/keratin electrospun nanofiber scaffold and microfiber scaffold, respectively. In a co-culture of keratinocytes and fibroblasts using a multi-layered scaffold, the two cells formed the epidermis and dermal layer on the PCL/keratin scaffold without territorial invasion. In the animal study, the PCL/keratin scaffold caused a faster regeneration of new skin without scar formation compared to the PCL scaffold. Our study showed that PCL/keratin scaffolds co-cultured with keratinocytes and fibroblasts promoted the regeneration of the epidermal and dermal layers in deep skin defects. Such finding suggests a new possibility for artificial skin production using multiple cells.

## 1. Introduction

During the skin wound-healing process, well-organized granulation tissue formation in the dermis and re-epithelization of the epidermis are essential. The crucial factor for granulation tissue formation is the proper infiltration of inflammatory cells and fibroblasts, while the crucial factor for re-epithelization is the proper migration and proliferation of keratinocytes [1,2]. Proper contraction of the wound is essential for restoring the dermal barrier; however, uncontrolled extreme contracture caused by excessive migration of fibroblasts and infiltration of disorganized collagen can lead to increased scar formation [3,4,5].

When the wound size is large, re-approximation of the skin, which is essential for re-epithelialization, is more difficult [3]. The deeper the wound, the more progenitor fibroblast stem cells, that initiate re-epithelialization from the wound margin, are destroyed [3]. Thus, larger and deeper wounds are associated with delayed wound healing and increased scar or wound contracture [3]. Artificial skin substitutes are suggested as a way to minimize these scars or wound contractures. Suitable skin substitutes should thus be fabricated considering the skin tissue anatomy [6] and wound-healing process [7].

Recently, multi-layered skin substitutes that mimic the genuine skin anatomy of multiple layers (epidermis, dermis, and hypodermis) have received attention. The epidermis mainly consists of keratinocytes and keratin-based extracellular matrix (ECM), while the dermis consists of fibroblasts and ECM (e.g., collagen, fibronectin, and elastin) produced by fibroblasts [8]. The nanofibers produced by electrospinning have many advantages for producing scaffolds as electrospun nanofibers can mimic the nanoscale structural dimensions of ECM, which consist of proteoglycan and fibrous proteins [9].

Polycaprolactone (PCL) is widely used as an electrospun material for tissue regeneration because of its superior biodegradability, biocompatibility, and proper mechanical properties [10,11,12]. However, PCL is a hydrophobic material that is deficient in cell recognition sites, which decreases cell affinity and limits cell adhesion [13,14]. Therefore, natural polymers, such as collagen, gelatin, hyaluronan, keratin, and fibrinogen, are blended into PCL to improve cell adhesion or affinity [15,16,17].

Meanwhile, keratin separated from hair and wool fibers has cell adhesion sequences, such as Arg-Gly-Asp (RGD) and leucine–aspartic acid–valine (LDV), which mainly exist in ECM proteins, such as fibronectin [18,19]. These peptide sequences promote cell attachment and proliferation [20]. Keratin is known to induce the proliferation of various cells and to increase cell adhesion. Keratin promotes adhesion and proliferation of fibroblasts more than type I collagen [20]. Moreover, alginate/keratin hydrogels have been found to induce more fibroblast growth and migration than alginate hydrogels [21]. During skin wound healing, keratins, which are made by keratinocytes, are one of the first waves of alarm. Keratins provide a structure for cell anchoring and regulate the proliferation or differentiation of keratinocytes [22]. Thus, keratin supplementation or scaffolds containing keratin have received great interest as a new biomaterial for skin regeneration [23,24]. Natural polymers, such as collagen, gelatin, hyaluronan, and fibrinogen, have limitations for large-scale production due to limited protein sources and the high cost of protein extraction [25]. Human hair waste has the potential to solve these problems as a keratin source, and several studies have shown that human hair keratin could be used as a scaffold for tissue regeneration [26,27,28].

In this study, we fabricated a PCL/keratin electrospun scaffold and cultured two cell layers of keratinocytes and fibroblasts on the electrospun scaffold to mimic the epidermis and dermis layer of the skin. Keratin was extracted from the human hair waste. To modulate the proper layers of keratinocytes and fibroblasts, we used two sizes of fibers for the scaffold: nanofibers for keratinocyte growth and microfibers for the growth of fibroblasts. Between the two layers, we added a 3D-printed support structure with PCL for easy handling of the cell-cultured scaffold and proper application on the wound site without unwanted folding or tearing. Using an animal deep skin defect model, we evaluated the effect of keratinocyte/fibroblast-cultured PCL/keratin electrospun scaffolds on skin regeneration and compared the effect of skin regeneration with keratinocyte/fibroblast-cultured PCL electrospun scaffolds.

## 2. Materials and Methods

### 2.1. Materials

Human hair was collected from local barber shops. PCL (Mn 45,000), urea (CH_4_N_2_O), sodium dodecyl sulfate (SDS), and 2-mercaptoethanol were purchased from Sigma-Aldrich (St. Louis, MO, USA). Sodium hydroxide and formic acid were purchased from Samchun Chemicals (Seoul, Korea). 

### 2.2. Extraction of Human Hair Keratin

Keratin was extracted from human hair according to a previously reported method [18,29,30]. Human hair was obtained from natural, undyed male hair in a barber shop, regardless of age. All the study were approved by the Animal Subjects Committee of Gachon University (Approval number: LCDI-2019-0105). Random hair samples were first washed with soap, followed by 70% ethanol to remove surface oils, rinsed extensively with water, dried, and then cut into short pieces. The defatted human hair (10 g) was mixed with distilled water (300 mL), urea (90 g), SDS (9.6 g), and 2-mercaptoethanol (9.6 g) in a 500 mL round-bottom flask. The mixed solution was adjusted to pH 9 with 1 mol/L sodium hydroxide and stirred for 48 h at 70 °C. The resulting mixture was centrifuged for 10 min at 8000 rpm, and the supernatant was filtered. The obtained solution was dialyzed against deionized water containing 0.1% 2-mercaptoethanol. The dialysate was replaced every 12 h, and dialysis was stopped after 84 h.

The resulting mixture was filtered using medical gauze to remove insoluble hairs. Keratin extracts were then centrifuged at 6000 rpm for 15 min. The resulting supernatant was dialyzed against distilled water for 72 h at room temperature using a cellulose tube (MWCO: 12,000–14,000 Da; SPL Life Science, Pocheon, Korea) by changing the outer solution every day. The dialysate was centrifuged again at 14,000 rpm for 25 min to remove the aggregated structures. Finally, the dialysate was poured into a flat-bottom flask, frozen at −86 °C, and lyophilized for 48 h to obtain keratin in powder form.

### 2.3. Preparation of Polymer Solution

PCL solutions of 20% (*w*/*v*) were prepared by dissolving PCL powder in formic acid with magnetic stirring for 2 h at room temperature. To prepare a 40% (*w*/*v*) PCL/keratin blend solution, keratinic extract powder (1.0 g) was dissolved in 5 mL of formic acid under continuous magnetic stirring at 40 °C for approximately 2 h, after which 1 g of PCL powder was dissolved in the solution.

### 2.4. Electrospinning Process

Electrospinning was performed using a horizontal electrospinning setup (NanoNC, Seoul, Korea). The syringe tip was positioned approximately 10 cm above the flat metallic platform. A voltage of 15–20 kV was used to charge the solution. The solution was dispensed from a single nozzle spinneret of 25 gauzes (NanoNC, Seoul, Korea) at a constant feed rate of 0.2 mL/h at 22 °C and humidity of 12.5% ± 2.5%. Fiber morphologies in scaffolds were observed using an optical microscope (Optical microscope, OPTIKA, Ponteranica, Italy), then fiber diameters and pore sizes were measured by an image software (ImageView Ver.3.7, Touptek Photonics, Hangzhou, China). The average values of fiber diameter and pore size were calculated by measuring five locations on the image and averaging them.

### 2.5. 3D Printing Process

A 3D model of the scaffold was constructed using Pro/Engineer 5.0 (PTC, Boston, MA, USA), and then exported as an STL file. The STL files were transferred to a 3D Bio-printer (Geo Technology, Incheon, Korea), and the printing process was carried out. The optimized printing parameters were as follows: nozzle diameter, 250 μm; printing temperature, 90 °C; speed, 300 mm/s; layer thickness, 200 μm.

### 2.6. Detection of Loaded Keratin in the Electrospun Fibrous Membrane

After fabrication of the multi-layer scaffold, an experiment was conducted to confirm the presence of keratin. The attenuated total reflection (ATF) mode of FT-IR (Nicolet iS5, Thermo, Waltham, MA, USA) was used to analyze and confirm the content of keratin in the scaffold. The spectrum of each sample was scanned 16 times at a resolution of 4 cm^−1^. Thereafter, the data were obtained.

### 2.7. Analysis of In Vitro Keratin Stability 

The stability of the contained keratin was evaluated by a weight change of the electrospun scaffold in phosphate buffered saline (PBS; Gibco, Waltham, MA, USA) for 1, 3, 5, 7, 14, and 30 days at a temperature of 37 °C, and then drying and weighing the scaffolds of each day. The stability value was shown as the scaffold weight percentage (%) using the following equation:

(1) $$\mathrm{Scaffold}\;\mathrm{weight}\;\left(\%\right)\;=\;\frac{W_{2}}{W_{1}}\times 100$$

where, *W*_1_ and *W*_2_ are the weights before (day 0) and after (each day) the precipitation of the scaffold, respectively. All experiments were conducted using three samples.

### 2.8. Cell Culture

Normal human dermal fibroblasts (HDFs) and human immortalized keratinocytes (HaCaT) were purchased from PromoCell (Heidelberg, Germany). HDFs were cultured in fibroblast growth medium (Gibco, Waltham, MA, USA) with 1% antibiotics, while HaCaT cells were cultured in Dulbecco’s Modified Eagle Medium (Gibco, Waltham, MA, USA) supplemented with 10% FBS (Gibco, Waltham, MA, USA) and 1% antibiotics at 37 °C in a 5% CO_2_ atmosphere.

### 2.9. Cell Viability

To evaluate the cytotoxicity of the developed scaffold, cell culture was performed. Before cell culture, a 5 mm diameter punch was used to create a scaffold in a disk shape. Each punched scaffold was transferred to 96-well plates (Corning, NY, USA) and HaCaTs or NHDFs were seeded at a density of 1.5 × 10^4^ cells/scaffold. After culturing for 1, 3, 5, and 7 days, a live/dead viability kit (Life Technologies, Carlsbad, CA, USA) was used to evaluate cell attachment and viability on electrospun ECM. Electrospun ECM disks were washed with ethanol and distilled water and incubated with 2 mM ethidium homodier-1 (EthD-1) (20 μL) and 5 mM calcein-AM (5 μL) in PBS for 20 min. Disks were viewed using a confocal microscope (Zeiss LSM 710, Oberkochen, Germany).

### 2.10. Cell Proliferation

To evaluate cell proliferation in the developed scaffold, cell culture was performed. Before cell culture, a 5 mm diameter punch was used to create a scaffold in a disk shape. Each punched scaffold was transferred to 96-well plates (Corning, NY, USA) and keratinocytes or fibroblasts were seeded at a density of 1.5 × 10^4^ cells/scaffold. At 1, 3, 5, and 7 days after culturing in 100 mL of growth medium with 10 μL of Cell Counting Kit 8 (CCK8, Dojindo, Kumamoto, Japan) for 2 h, the cell proliferation rate was determined by measuring the absorbance at an optical density of 450 nm using a microplate reader (VERSAmax, San Jose, CA, USA).

### 2.11. Immunocytochemistry (ICC)

To evaluate the culturing performance and layer-arrangement of NHDFs and HaCaTs, after progressing co-culture of the two cells on the scaffold, immunofluorescence staining using anti-fibronectin (FN, Abcam, Cambridge, UK), a marker specific for fibroblasts, and anti-cytokeratin-14 (CK14, Abcam, Cambridge, UK), a marker for identifying keratinocytes, was performed. After fixation of the cultured scaffolds with 4% paraformaldehyde (PFA, Biosesang, Seongnam, Korea), blocking was achieved by incubating the cells for 60 min in 1% normal goat serum (Vector laboratories, Burlingame, CA, USA) in PBS/0.3% Triton X-100 (Sigma, St. Louis, MO, USA). The cells were incubated overnight at 4 °C with primary antibodies (Abcam, Cambridge, UK) against fibronectin or cytokeratin-14 at a dilution of 1:200. The cells were then labeled with secondary antibodies of donkey anti-mouse IgG H&L (anti-mouse, Abcam, Cambridge, UK) or donkey anti-rabbit IgG H&L (anti-rabbit, Abcam, Cambridge, UK) at a dilution of 1:500 for 1 h at 37 °C. After washing with Tris-buffered saline, twice, the scaffolds were counterstained with 4′,6-diamidino-2-phenylindole (DAPI, Sigma, St. Louis, MO, USA) and images were acquired using a confocal microscope (LSM-710, Zeiss, Oberkochen, Germany).

### 2.12. In Vivo Wound Healing Study

To evaluate the skin regeneration ability of the developed scaffold, the change in the wound area over time was observed after implanting the scaffold into the nude mouse. Six-week-old male immuno-deficient nude mice (*n* = 12) (BALB/c, OrientBio, Seongnam, Korea) were randomly assigned to two groups (PCL and PCL/keratin groups). After co-culturing keratinocytes and fibroblasts for 4 days before implantation, initial wounds were created by a skin punch with 5 mm diameter at the backside of the mouse, and then co-cultured scaffolds were implanted. After sacrificing the mice at days 7 and 14, wound tissues were fixed in 4% PFA, and then sectioned into a 4 μm thick paraffin block and stained. Snapshot images were acquired at days 0, 3, 7, and 14 from the implanted area. To calculate the wound area, ImageJ (ver. 1.43u, National Institute of Health, Bethesda, MD, USA) was used. The study protocol was approved by the Animal Subjects Committee of Gachon University (Approval number: LCDI-2019-0105).

### 2.13. Hematoxylin and Eosin (H&E) Staining

After deparaffinization, slices were placed in hematoxylin (Sigma, St. Louis, MO, USA) for 5 min, washed with DI water, and dipped into 1% acid alcohol (HCl + 70% EtOH) for 10 s. After washing with DI water, the slices were dipped in eosin (Sigma, St. Louis, MO, USA) for 3 min, washed with DI water, and dipped into ammonia for 10 s. Subsequently, they were washed with DI water and dehydrated using 70%, 80%, 90%, and 100% ethanol (Sigma, St. Louis, MO, USA) and xylene (Sigma, St. Louis, MO, USA).

### 2.14. Masson’s Trichrome Staining

After deparaffinization, the sample slices were dipped into Bouin’s solution (Sigma, St. Louis, MO, USA) and incubated for 1 h at 56 °C. Thereafter, they were washed thoroughly with de-ionized water for 10 min and dipped into scarlet solution (Sigma, St. Louis, MO, USA), phosphomolybdic–phosphotungstic acid (phosphomolybdic acid, Sigma, St. Louis, MO, USA; phosphotungstic acid, Sigma), and aniline blue solution (M5528-25G, Sigma, St. Louis, MO, USA) for 10 min each. The slices were also washed with DI water and dipped into acetic acid (Sigma, St. Louis, MO, USA) for 3 min and hematoxylin for 10 min. Finally, these slices were washed again with deionized water and dehydrated.

### 2.15. Statistical Analysis

All experiments were performed in triplicate, and the representative or average data are presented, unless otherwise stated. The data were analyzed using Prism (ver. 7; GraphPad sofware, San Diego, CA, USA) software. The data within a given group or between groups were compared using one-way analysis of variance (ANOVA). Significant differences were defined as * *p* < 0.05 and ** *p* < 0.01.

## 3. Results

### 3.1. Characterization of the Electrospun Fibrous Membrane

**Figure 1:**
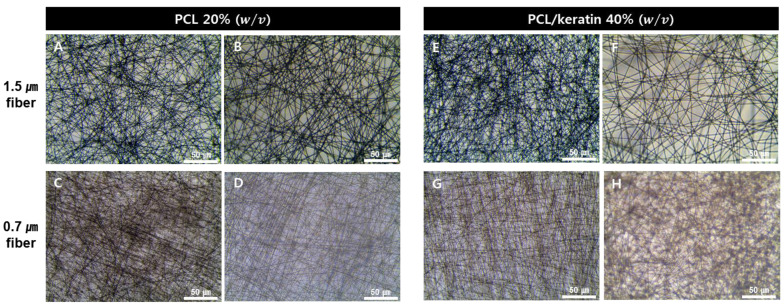
Electrospun layers with and without keratin. (**A**,**B**) PCL scaffold with 1.5 μm fibers (7 kV). (**C**,**D**) PCL scaffold with 0.7 μm fibers (20 kV). (**E**,**F**) PCL/keratin scaffold with 1.5 μm fibers (7 kV). (**G**,**H**) PCL/keratin scaffold with 0.7 μm fibers (21 kV).

In this study, keratin extracted from human hair was electrospun to fabricate micro-fibrous and nanofibrous mats that mimic the epidermis and dermis of human skin. The fiber diameters of the PCL and PCL/keratin fibers and the electrospinning process parameters are listed in Table 1. Figure 1 shows images of the electrospun PCL and PCL/keratin fibers captured with an optical microscope.

The fiber diameter of the microfibers made with PCL was 1.5 ± 0.1 μm and the pore size was 6.2 ± 2 μm. The fiber diameter of nanofibers made with PCL was 0.7 ± 0.1 μm and the pore size was 4.5 ± 1 μm. The fiber diameter of microfibers made with PCL/keratin was 1.7 ± 0.2 μm and the pore size was 6.4 ± 1 μm. The fiber diameter of nanofibers made with PCL/keratin was 0.7 ± 0.1 μm and the pore size was 4.8 ± 1 μm. Furthermore, the fabricated electrospun fibers had a regular linear shape with no anomalous conformations, such as ribbon-like structures or bead shapes.

### 3.2. Stability Measurement of a Keratin-Loaded Electrospun Fibrous Membrane

**Figure 2:**
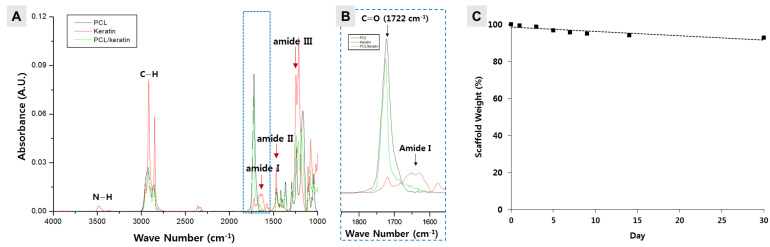
Analysis of PCL/keratin. (**A**) FT-IR spectra analysis, (**B**) zoom graph section of FT-IR, and (**C**) weight measurement of PCL/keratin 40% (*w*/*v*) membranes.

The goal of this study was to determine whether keratin-containing electrospun membranes assist in skin tissue regeneration. It was necessary to confirm the presence of keratin in the membrane produced through electrospinning using a PCL/keratin solution. Thus, the presence of keratin was measured using FT-IR spectrum analysis of the fabricated specimen. As shown in Figure 2A, from the FT-IR spectrum, amide I (1600–1700 cm^−1^), amide II (1480–1580 cm^−1^), and amide III (1220–1300 cm^−1^) peaks, depicting the presence of keratin, could be observed. Especially, as shown in Figure 2B, which is a zoom graph section of FT-IR, amide I peak (red line) was observed near 1650 cm^−1^. This was a peak not found in PCL (black line), indicating that the extracted material was keratin.

The main promoter of polymer degradation in the body is the aqueous environment of the tissue. Thus, we evaluated the degradation of the PCL/keratin scaffold in PBS solution at 37 °C for up to 30 days. As shown in Figure 2C, the PBS solution showed a low decomposition rate. When immersed in PBS for a week, the FT-IR spectrum showed that the membrane still had keratin (Figure S1). In the first week, 4% of the total weight of the PCL/keratin scaffold was lost, and the weight loss of the scaffold was less than 8% at the end of the experiment (day 30). 

### 3.3. Cyto-Compatibility of the Scaffolds

#### 3.3.1. Effect of Fiber Diameter on Cell Viability

**Figure 3:**
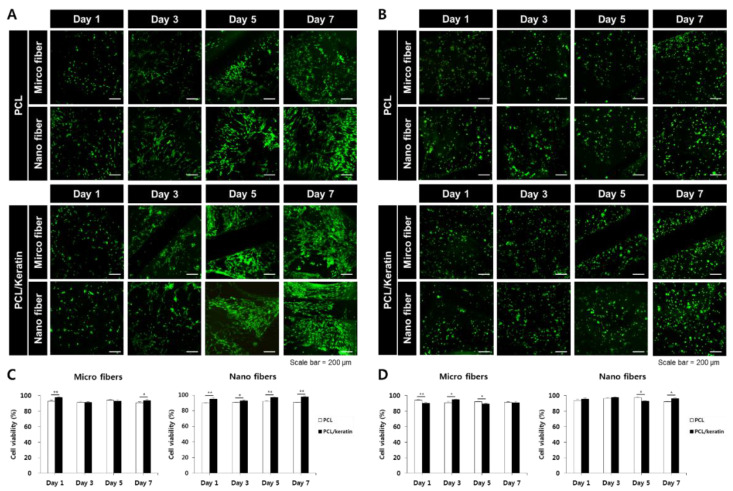
Viability of NHDFs (**A**,**C**) and HaCaTs (**B**,**D**) cells cultured on PCL and PCL/keratin electrospun scaffolds, measured via live/dead assay (* *p* < 0.05, ** *p* < 0.01, Scale bar = 200 μm).

**Figure 4:**
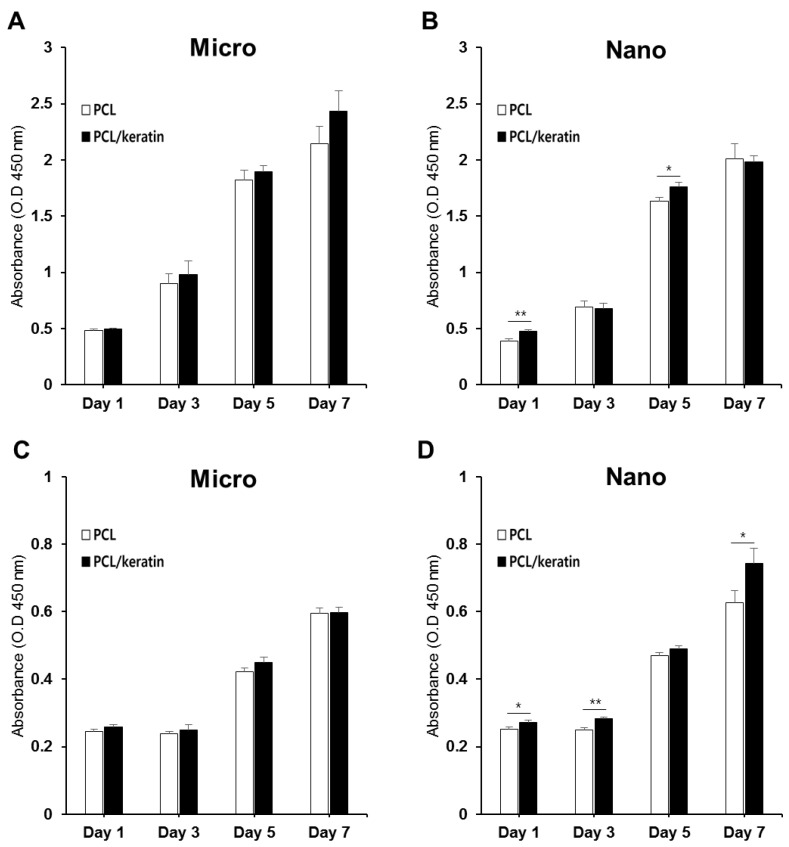
Comparison of the cell proliferation results. (**A**) NHDFs on microfibers, (**B**) NHDFs with nanofibers, (**C**) HaCaTs on microfibers, and (**D**) HaCaTs on nanofibers (* *p* < 0.05, ** *p* < 0.01).

The cytotoxic profile of the electrospun scaffold was analyzed via a live/dead assay over 1, 3, 5, and 7 days. As shown in Figure 3A,B, the live/dead assay of NHDF and HaCaT cells at 1, 3, 5, and 7 days revealed no toxic effect on the present materials. In addition, as shown in Figure 4C,D, approximately 90% of NHDF and HaCaT cells lived during the total culture time (7 days) without any toxic effects.

#### 3.3.2. Effect of Fiber Diameter on Cell Proliferation

As keratin fibers might not be cytotoxic to the cells tested herein, cell adhesion and spreading might be an important contributing factor to scaffolds for tissue engineering. The effect of PCL and PCL/keratin on cell attachment and proliferation was evaluated using the CCK-8 assay. We also analyzed the effect of micro- and nano-environments of the fibrous scaffold on NHDF and HaCaT adhesion and proliferation.

Figure 4 shows the in vitro proliferation of NHDF and HaCaT cells cultured on PCL and PCL/keratin scaffolds for 1, 3, 5, and 7 days, as evaluated by a CCK-8 assay. The cells cultured on the electrospun fibrous scaffolds gradually proliferated for up to 7 days. On day 1, no significant differences were observed in the cell proliferation rates. However, after 7 days of culture, there was a noticeable difference in the morphology of the scaffolds, which each cell favored. NHDFs showed higher proliferation rates in microfibers and HaCaTs showed higher proliferation rates in nanofibers.

### 3.4. Fabrication of the Multi-Layer Hybrid Scaffolds

**Figure 5:**
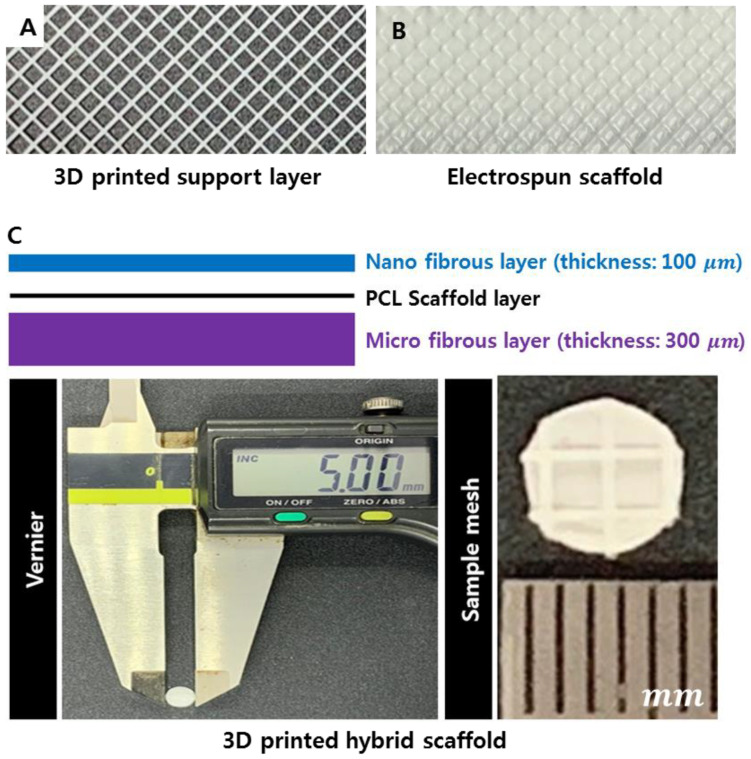
Fabrication results of the multi-layer scaffolds. (**A**) Optical image of the 3D-printed support layer, (**B**) optical image of the electrospun and 3D-printed scaffold, and (**C**) optical image of the three-layer electrospinning 3D-printed hybrid scaffold.

To culture NHDF and HaCaT cells, we fabricated 3 three-layered scaffolds: the upper layer consisted of 100 µm thick nanofibers for the culture of HaCaT cells, the middle layer was a 3D-printed support layer, and the lower layer consisted of 300 µm thick microfibers for the culture of NHDF cells (Figure 5).

### 3.5. Co-Culture of the Multi-Layer Scaffolds

**Figure 6:**
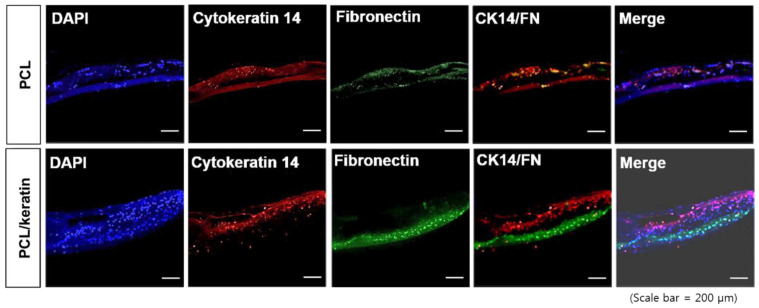
Immunocytochemistry results of the co-cultured scaffolds. (Scale bar = 200 μm). HaCaT cells were seeded 3 days (72 h) after NHDF seeding. On day 4 (96 h) after NHDF seeding, the HaCaT created a layer, which was stained by cytokeratin-14. Beneath the HaCaT layer, the NHDF produced a layer stained by fibronectin.

A co-culture of NHDF and HaCaT cells was performed on the three-layered scaffold. Three days after NHDF seeding on the micro-fibrous layer, HaCaT cells were seeded on the nanofibrous layer by flipping over the scaffold (Figure 6). Four days after NHDF seeding, the NHDF layer created a layer that was stained with cytokeratin-14. On the other side of the NHDF layer, the HaCaT layer was stained with fibronectin. Keratinocytes (HaCaTs), which were stained by cytokeratin-14, formed thicker layers in the nanofibrous layer of the PCL/keratin multi-layer scaffold compared with the PCL multi-layer scaffold. Fibroblasts (NHDFs) formed a thick layer in the micro-fibrous layer of the PCL/keratin multi-layer scaffold; however, fibroblasts in the microfiber layer of the PCL multi-layer scaffold barely formed a layer. Two layers consisting of keratinocytes and fibroblasts in the PCL/keratin multi-layer scaffold were well-separated, and the fibroblasts did not infiltrate into the upper nanofibrous layer.

### 3.6. In Vivo Wound-Healing Study

**Figure 7:**
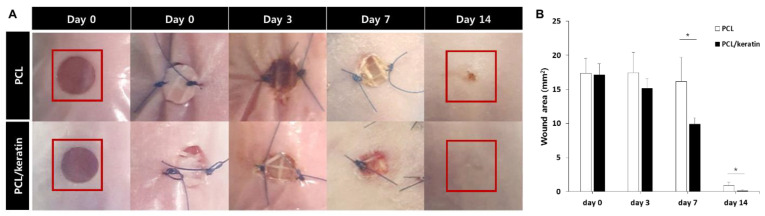
In vivo wound-healing results of PCL and PCL/keratin scaffolds. (**A**) Optical images of the wound areas of mice, and (**B**) analysis of the wound-healing performances using the PCL and PCL/keratin scaffolds (* *p* < 0.05).

After implantation of the scaffolds in nude mice, most of the wound areas were filled with new skin tissue in either the PCL/keratin multi-layer scaffold implantation group (PCL/keratin group) or PCL multi-layer scaffold implantation group (PCL group) at 2 weeks. However, the scar remained in the PCL group, but the PCL/keratin group healed with a smooth surface (Figure 7A). In addition, from the analysis of wound areas over two weeks, the healing speed in the PCL/keratin group was significantly faster than that of the PCL group on days 7 and 14 (Figure 7B).

**Figure 8:**
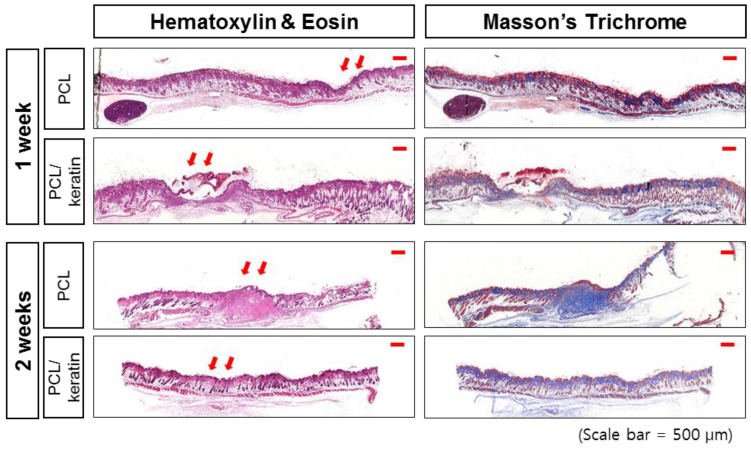
Staining results of the implanted PCL and PCL/keratin scaffolds.

The amount of epithelialization was evaluated using H&E staining. The epidermis was easily identified in all images, showing the presence of polarized basal keratinocytes, a spinous layer composed of cuboidal, differentiating keratinocytes, a granular cell layer, and finally the outermost stratum corneum composed of flattened dead cells (Figure 8). The dermis layers, mainly the papillary layer adjacent to the epidermis, and the reticular layer, denoted by a greater concentration of thick collagen fiber cells and bundles, were also evident.

The production of collagen in the dermal layer is important for skin strengthening. Thus, collagen deposition was evaluated using Masson’s trichrome stain. The epidermis and dermis were easily recognized in the immunostaining of collagen-I in Figure 8 for all treated wounds and the control wound. However, the control wound was poor in granulated tissue, and no hair follicles or sebaceous glands were observed. In addition, a high concentration of fibroblasts and weak formation of type I collagen fibers were observed. These features indicate that the control wound was in an early proliferative stage, even though the wound appeared closed and fully epithelialized.

## 4. Discussion

Both cell proliferation and organization are essential for the proper healing of deep skin wounds. The ideal scaffold for skin regeneration should (1) provide an instant cover for the wound, (2) inhibit wound infection, (3) accelerate wound closure, (4) guarantee the proliferation of various types of cells, and (5) assist proper arrangement of these cells for regeneration and the prevention of scar formation. Thus, multi-layered scaffolds are recognized to have potential as scaffolds for skin regeneration by accelerating wound closure and rebuilding the original histological structure of the skin [31]. In our study, we fabricated a multi-layered scaffold with PCL and keratin for skin wound regeneration.

Keratin is an insoluble protein that mainly exists in epithelial tissues and can be extracted from feathers, nails, hooves, wool, and human hair [32,33]. Keratins can self-assemble and be crosslinked to produce porous or fibrous scaffolds [32,34,35,36,37]. In particular, keratin is widely used as a wound dressing or scaffold for skin regeneration [38], as keratin promotes cell adhesion or the proliferation of keratinocytes or fibroblasts [20,21]. Although keratin has poor mechanical properties, various studies have been conducted to improve mechanical strength, while having good cell affinity, through mixing with various biomaterials [39,40,41,42,43].

Electrospinning is a useful method for the fabrication of nanofiber membranes with various polymers [44]. Electrospun membranes have high porosity, which increases the permeability of oxygen and nutrition [45]. Nanofibers have a high surface-to-volume ratio and possess features similar to those of the natural ECM of skin, thereby leading to increased cell adhesion, migration, and proliferation [45]. During electrospinning, the mixing ratio of keratin and the polymer affects the viscosity and conductivity [46]. By increasing the keratin content, the viscosity of the polymer decreases, and the conductivity increases [46]. Polymer contents higher than 10 wt% are known to be sufficient for avoiding the breakage of the electrically driven jet [47]. In the case of PCL, since it is a synthetic polymer with hydrophobicity, when an electrospinning membrane is manufactured using a PCL/keratin mixture, the cell affinity increases as the amount of keratin increases. However, a high content of keratin could produce droplets or beaded nanofibers [48]. Electrospinning with 100% keratin can produce ribbon-like anomalous conformations [49]. In our experiment, as the content of keratin increased, it was difficult to make nanofibers. Therefore, in this study, the optimal keratin ratio (PCL:keratinic extract = 50:50) condition for stably producing nano/microfibers within the specifications of our electrospinning system was secured. At that ratio, no bead or ribbon-like conformation was found during the electrospinning of the nanofibers and microfibers.

Although keratin promotes cell adhesion or the proliferation of keratinocytes or fibroblasts [20,21], if keratin is lost during the electrospinning process, the intended performance may not be achieved. The presence of keratin was measured through FT-IR spectrum analysis of the fabricated electrospun fiber, and amide I (1600–1700 cm^−1^), amide II (1480–1580 cm^−1^), and amide representing keratin III (1220–1300 cm^−1^) peaks were observed in the spectrum. This result revealed that keratin was safely settled in the fiber during the manufacturing process of the electrospun fibers.

The stable inclusion of keratin in the electrospun fibers during long-term cell culture is an important factor for achieving the purpose of this study. To confirm this inclusion, the weight lost over time was measured after immersing the electrospun scaffolds in PBS. The electrospun scaffolds showed only 8% weight loss in PBS for 30 days; however, they did not degrade linearly over time. The degradation of keratin may be accelerated by various enzymes distributed in the body under the skin of a mouse. However, the high stability of keratin in the long-term in vitro experiment for 30 days might indirectly prove the stability of keratin in an animal for 2 weeks.

The local geometry of the scaffold, such as porosity, pore size, fiber diameter, and local compliance, is important in interactions between cells and scaffolds as the cells sense those local geometry features [50]. Thus, proper designing of the local geometry depending on the cell type of the target tissue is essential [51,52]. In our study, keratinocytes showed superior proliferation on nanofibers, while fibroblasts showed superior proliferation on microfibers. In addition, the proliferation of both keratinocytes and fibroblasts increased on the keratin-containing scaffold compared to the pure PCL scaffold.

To create a multi-layer scaffold, we fabricated nanofibers as the upper layer for keratinocyte cultivation, and fabricated microfibers as the lower layer for fibroblast cultivation. It is a well-known concept that resemblance to histological or anatomical structures leads to similar physiological functions [53]. Accordingly, many researchers have produced multilayer artificial skins with multiple layers of the epidermis, dermis, and hypodermis [31]. In our study, we mimicked the epidermis by fabricating nanofiber layers of 100 μm thickness, with keratinocytes cultured on this layer, and the dermis by fabricating 300 μm thick nanofiber layers in which fibroblasts were cultured. Fibroblasts must infiltrate into the scaffold where the fibroblasts are assigned to regenerate the proper dermal layer; however, fibroblast infiltration should not progress to the epidermal layer. The pore size or porosity is a determining factor for cell migration into the scaffold [54]. In our study, the pore size of the nanofiber layer ranged from 4.52 to 4.82 μm. We believe that these pore sizes could inhibit the infiltration of fibroblasts into the nanofiber layer. The cross-sectional area of the multi-layer scaffold after cell culture of keratinocytes and fibroblasts showed that fibroblasts did not infiltrate the nanofiber layers. Furthermore, the keratinocytes and fibroblasts were found to more efficiently proliferate and form distinctively different layers on the PCL/keratin scaffold than on the PCL scaffold. Keratinocytes on the PCL/keratin scaffold formed a thicker layer on the upper nanofiber layer than on the PCL scaffold. Moreover, fibroblasts on the PCL scaffold did not have a sufficient layer thickness compared with those on the PCL/keratin scaffold.

A multi-layer scaffold that mimics the histological structure of skin is known to induce proper healing of damaged skin by stimulating the regeneration of the dermis and re-epithelialization [55,56]. In our study, we observed that wound closure was markedly faster when the wound was covered with the PCL/keratin scaffold with cultured keratinocytes and fibroblasts than the pure PCL scaffold with cultured keratinocytes and fibroblasts. Based on histological examination, epithelialization and keratinocyte proliferation in the epidermis were superior in the PCL/keratin scaffold relative to the PCL scaffold. The proliferation of fibroblasts on PCL/keratin was also superior to that on PCL scaffolds. Further, the collagen deposition of the PCL/keratin scaffold was superior to that of the PCL scaffold. The keratinocytes and fibroblasts cultured on multi-layer PCL/keratin scaffolds appeared to cause better skin wound healing than keratinocytes and fibroblasts cultured on multi-layer PCL scaffolds.

## 5. Conclusions

In this study, a skin substitute was developed to reconstruct a multi-layer of the epidermis and dermis by combining optimized electrospun nano- and micro-sized fibers. By incorporating human keratin into the skin substitute, we provided a more suitable 3D environment for keratinocytes and fibroblasts. After co-culture of keratinocytes and fibroblasts on the multi-layer PCL/keratin scaffold, we used the skin substitute for wound healing in animals. This scaffold was found to result in better wound healing than the PCL-only scaffold. The developed scaffold with multiple cells suggests a new possibility for the regeneration of the epidermal and dermal layers for deep skin defects.

## Figures and Tables

**Table 1:** Fiber diameters and pore sizes of 20% (*w*/*v*) PCL and 40% (*w*/*v*) PCL/keratin with respect to the electrospinning process parameters.

	Fiber Diameter (μm)	Pore Size (μm)	Electrospinning Condition			
			Concentration (*w*/*v*%)	Voltage (kV)	Flow Rate (mL/h)	Distance (mm)
**PCL-Micro**	1.5 ± 0.1	6.2 ± 2	20	7	0.2	100
**PCL-Nano**	0.7 ± 0.1	4.5 ± 1	20	20		
**PCL/keratin-Micro**	1.7 ± 0.2	6.4 ± 1	40	7		
**PCL/keratin-Nano**	0.7 ± 0.1	4.8 ± 1	40	21		

Temperature: 22.6 ± 0.1 °C, Humidity: 12.5 ± 3%.

## Data Availability

Data is contained within the article.

## Supplementary Materials

The following are available online at https://www.mdpi.com/article/10.3390/polym13162584/s1, Figure S1: Analysis of the degradation of PCL/keratin membrane (Comparison between day 0 and day 7).
