# Supplementary material for: Development of a Multi-Layer Skin Substitute Using Human Hair Keratinic Extract-Based Hybrid 3D Printing
Source: Polymers (Basel). 2021 Aug 4;13(16):2584. doi: 10.3390/polym13162584 (PMC8401121; doi:10.3390/polym13162584)
Supplement: Supplementary file 1 [file polymers-13-02584-s001.zip › polymers-1292933-supplementary.pdf]

Development of a multi-layer skin substitute using human hair keratin-based hybrid 3D printing

< Supplementary Materials >

Won Seok Choi [1,†], Joo Hyun Kim [1,†], Chi Bum Ahn [2], Ji Hyun Lee [2], Yu Jin Kim [3], Kuk Hui Son [4,*] and Jin Woo Lee [1,2,*]

[1]Department of Health Sciences and Technology, GAIHST, Gachon University, Incheon, Republic of Korea

[2]Department of Molecular Medicine, College of Medicine, Gachon University, Incheon, Republic of Korea

[3]Department of Plastic and Reconstructive Surgery, Gachon University Gil Medical Center,College of Medicine, Gachon University,Incheon, Republic of Korea

[4]Department of Thoracic and Cardiovascular Surgery, Gachon University Gil Medical Center, Collegeof Medicine, Gachon University, Incheon, Republic of Korea

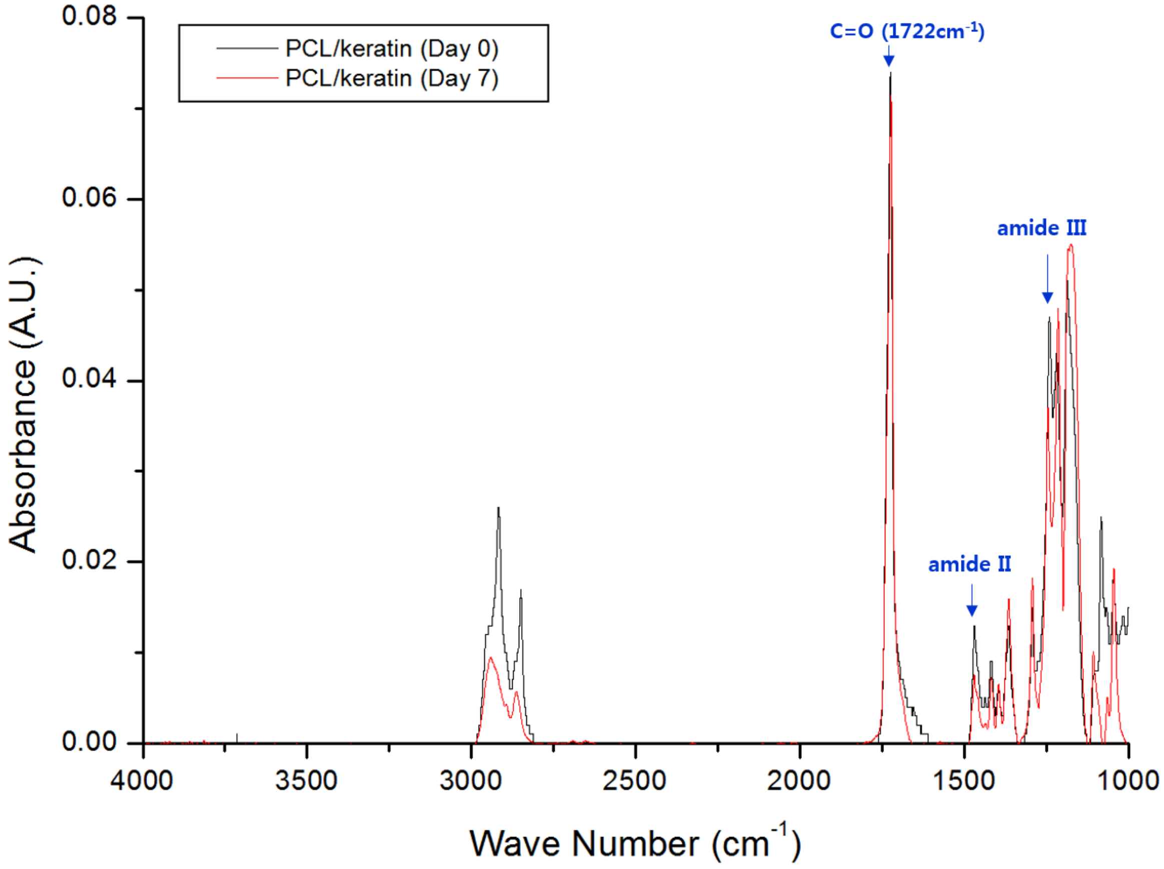

Figure S1 Analysis of the degradation of PCL/keratin membrane (Comparison between day 0 and day 7)
